# Distinct T cell responsiveness to different COVID-19 vaccines and cross-reactivity to SARS-CoV-2 variants with age and CMV status

**DOI:** 10.3389/fimmu.2024.1392477

**Published:** 2024-05-07

**Authors:** Jolanda Brummelman, Sara Suárez-Hernández, Lia de Rond, Marjan Bogaard-van Maurik, Petra Molenaar, Emma van Wijlen, Debbie Oomen, Lisa Beckers, Nynke Y. Rots, Josine van Beek, Mioara A. Nicolaie, Cécile A. C. M. van Els, Mardi C. Boer, Patricia Kaaijk, Anne-Marie Buisman, Jelle de Wit

**Affiliations:** ^1^ Center for Infectious Disease Control, Dutch National Institute for Public Health and the Environment (RIVM), Bilthoven, Netherlands; ^2^ Infectious Diseases and Immunology, Department of Biomolecular Health Sciences, Faculty of Veterinary Medicine, Utrecht University, Utrecht, Netherlands

**Keywords:** COVID-19, vaccine, mRNA, T cell response, CMV, age, cross-reactivity, VOCs

## Abstract

**Introduction:**

Accumulating evidence indicates the importance of T cell immunity in vaccination-induced protection against severe COVID-19 disease, especially against SARS-CoV-2 Variants-of-Concern (VOCs) that more readily escape from recognition by neutralizing antibodies. However, there is limited knowledge on the T cell responses across different age groups and the impact of CMV status after primary and booster vaccination with different vaccine combinations. Moreover, it remains unclear whether age has an effect on the ability of T cells to cross-react against VOCs.

**Methods:**

Therefore, we interrogated the Spike-specific T cell responses in healthy adults of the Dutch population across different ages, whom received different vaccine types for the primary series and/or booster vaccination, using IFNɣ ELISpot. Cells were stimulated with overlapping peptide pools of the ancestral Spike protein and different VOCs.

**Results:**

Robust Spike-specific T cell responses were detected in the vast majority of participants upon the primary vaccination series, regardless of the vaccine type (i.e. BNT162b2, mRNA-1273, ChAdOx1 nCoV-19, or Ad26.COV2.S). Clearly, in the 70+ age group, responses were overall lower and showed more variation compared to younger age groups. Only in CMV-seropositive older adults (>70y) there was a significant inverse relation of age with T cell responses. Although T cell responses increased in all age groups after booster vaccination, Spike-specific T cell frequencies remained lower in the 70+ age group. Regardless of age or CMV status, primary mRNA-1273 vaccination followed by BNT162b2 booster vaccination showed limited booster effect compared to the BNT162b2/BNT162b2 or BNT162b2/mRNA-1273 primary-booster regimen. A modest reduction in cross-reactivity to the Alpha, Delta and Omicron BA.1, but not the Beta or Gamma variant, was observed after primary vaccination.

**Discussion:**

Together, this study shows that age, CMV status, but also the primary-booster vaccination regimen influence the height of the vaccination-induced Spike-specific T cell response, but did not impact the VOC cross-reactivity.

## Introduction

In response to the SARS-CoV-2 pandemic, various vaccines have been offered to the general population of the Netherlands: the mRNA vaccines BNT162b2 (Pfizer/BioNTech) and mRNA-1273 (Moderna); and the viral-vector based vaccines ChAdOx1 nCoV-19 (AstraZeneca) and Ad26.COV2.S (Janssen). Booster vaccination was subsequently implemented to prolong vaccine impact, for which mRNA vaccines were employed.

Notably, vaccine effectiveness against infection wanes over time and vaccine protection against hospitalization was reported to remain stable for at least 6 months (1). Neutralizing Spike-specific antibodies are generally held responsible for preventing SARS-CoV-2 infection and limiting disease. However, antibody levels wane and, moreover, new Variants-of-Concern (VOCs) show escape mutations in the Spike protein, resulting in diminished virus neutralization. Importantly, several studies show that COVID-19 vaccination induces Spike-specific T cells (2–4). In contrast to induced antibodies, levels of Spike-specific T cells seem to be more stable over time and have been shown to be cross-reactive against most tested VOCs (3, 5–8). Moreover, SARS-CoV-2-specific T cells have been shown to be associated with limiting disease severity (9–11) and contribute to protection against hospitalization and death due to COVID-19 (6, 11, 12).

Older adults are a vulnerable group with regards to susceptibility and severity of COVID-19. In the Netherlands, around 90% of deceased patients during the COVID-19 pandemic was >70 years of age (13) and this age group comprised the majority of hospitalized individuals, despite a high vaccination coverage (93% for the primary series and 68% for booster vaccination) (14). Age-associated dysfunction of the immune system, referred to as immunosenescence, renders older adults more susceptible to infection by emerging viruses and less responsive to vaccination, which can be partly explained by a decline in T cell immunity (15). This age-related immune dysfunction has also been associated with persistent latent infections, as caused by cytomegalovirus (CMV), of which the hallmark is to induce immunosenescent CD28^-^ T cells that have been previously linked to increased risk of COVID-19 severity and hospitalization (16, 17). CMV incidence in the Netherlands ranges from 45% in younger adults to nearly 80% in the population >80 years old (18, 19), thus being a potential factor contributing to impaired T cell immunity against COVID-19 among older adults. In addition, induction of cross-reactive SARS-CoV-2-specific T cell responses by vaccination is critical for protection against severe disease and emerging VOCs in this age group in particular.

To date, it remains unclear what the role of age, CMV status, and the received primary-booster vaccine combination is on the Spike-specific T cell response to COVID-19 vaccination, and its cross-reactivity against VOC. Improved knowledge herein will help developing strategies to enhance protection in the vulnerable risk groups.

Here, we assessed Spike-specific T cell responses in a cohort of SARS-CoV-2 infection-naive adults (n=208) in the Dutch population across different age groups receiving different vaccine types for the primary series (BNT162b2, mRNA-1273, ChAdOx1 nCoV-19, or Ad26.COV2.S) and/or booster vaccination (BNT162b2 or mRNA-1273). Additionally, the influence of latent CMV infections herein was interrogated.

## Materials and methods

### Study population

**Figure 1:**
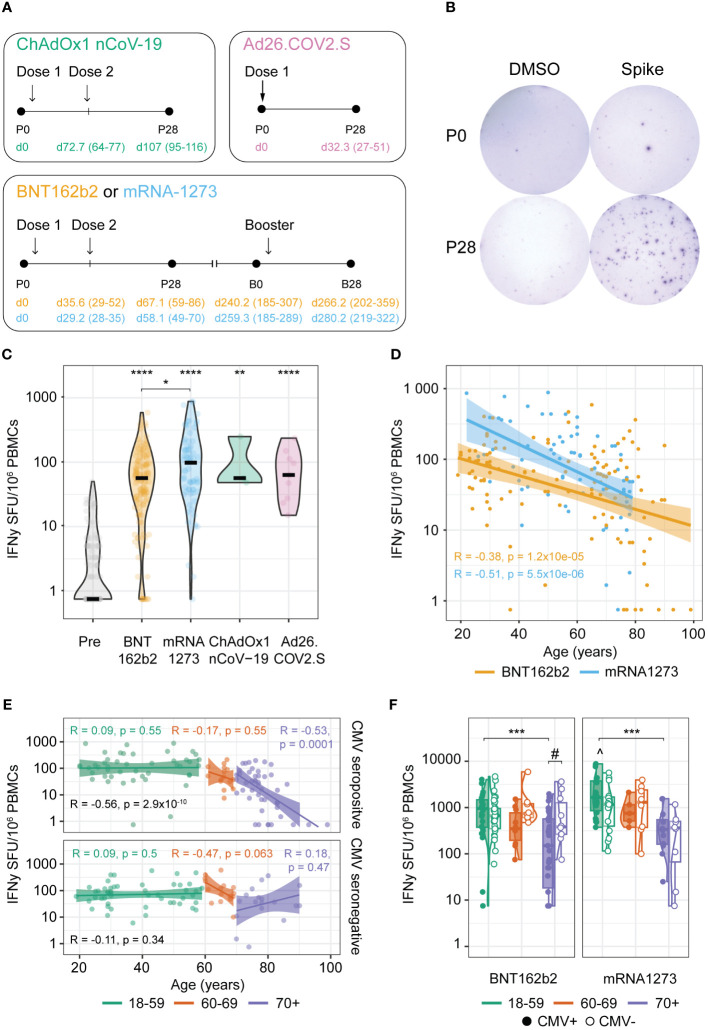
Age-dependent robust anti-Spike T cell responses induced by primary COVID-19 vaccination. **(A)** Schematic overview of the vaccination schemes of the tested cohorts. Numbers in parenthesis represent the range of days and colors represent the received vaccination. P0: pre-vaccination; P28: 28 days post-primary vaccination series; B0: pre-booster vaccination; B28: 28 days post-booster vaccination. **(B)** Representative wells of isolated PBMCs at timepoints P0 and P28 stimulated with DMSO (negative control) or an overlapping peptide pool of the ancestral vaccine strain Spike protein in an IFNγ T cell ELISpot assay. **(C–F)** IFNγ T cell response at timepoints P0 and P28. **(C)** Spike-specific T cell response at P0 and induced by the BNT162b2, mRNA-1273, ChAdOx1 nCoV-19, and Ad26.COV2.S vaccines at P28. *p<0.05, **p<0.01, ****p<0.0001 compared to P0 unless otherwise indicated; Kruskal-Wallis test and Dunn’s test with Benjamini-Hochberg adjustment for multiple comparisons. **(D, E)** Spearman correlation between age of the participants and Spike-specific IFNγ T cell response at P28; **(D)** Correlation coefficients (R) and p-values (p) are given for BNT162b2 and mRNA-1273 vaccinated participants; **(E)** Correlation coefficients and p-values are given for all according to cytomegalovirus (CMV) status (black) or stratified per age cohort (green: 18-59 years; orange: 60-69 years; purple: 70+ years). **(F)** Spike-specific IFNγ T cell response upon BNT162b2 or mRNA-1273 vaccination in different age groups stratified by CMV serostatus as indicated. ***p<0.001 among CMV-seropositive participants; #p<0.05; ^p<0.05 mRNA-1273 versus BNT162b2 among 18-59 CMV seropositive participants. Kruskal-Wallis test and Dunn’s test with Benjamin-Hochberg adjustment for multiple comparisons. SFU, Spot Forming Units.

Data from participants of three longitudinal SARS-CoV-2 vaccination studies within one framework with similar study design were combined. Two were observational COVID-19 vaccination cohort studies: one focusing on adults 18 to 60 years of age (NL76440.041.21, EudraCT: 2021-001357-31), and one on the ageing population over 60 years of age (NL74843.041.21, EudraCT: 2021-001976-40). In these studies, vaccines were rolled-out per age group from old to young according to the national vaccination campaign. For the primary vaccination series, vaccinees received BNT162b2 (Pfizer/BioNTech), mRNA-1273 (Moderna), ChAdOx1 nCoV-19 (AstraZeneca), or Ad26.COV2.S (Janssen) vaccines; and for booster vaccination either BNT162b2 or mRNA-1273 vaccines were given, as per national policy (**Figure 1A**). Due to limited number of participants vaccinated with ChAdOx1 nCoV-19 and Ad26.COV2.S, the majority of comparisons for statistical purposes have been performed on the BNT162b2 and mRNA-1273 vaccine groups.

The third study concerns an intervention study (Vital) investigating seasonal influenza and pneumococcal conjugate vaccine responses in older adults (≥65 years) compared with younger and middle-aged adults (25-49; 50-64 years). With a protocol amendment, vaccine responses to a primary series of mRNA-1273 followed by a booster dose with BNT162b2 have been added to the study (NL69701.041.19, EudraCT: 2019-000836-24) (**Figure 1A**). Ethical approval was obtained through the Medical Research Ethics Committee Utrecht in all three studies. All participants provided written informed consent. All trial-related activities were conducted according to Good Clinical Practice, including the provisions of the Declaration of Helsinki.

Samples from participants who experienced a SARS-CoV-2 infection prior or within 2 weeks after providing a post-vaccination sample were excluded from analysis in this study. Additionally, participants who experienced an infection in between the two doses of the BNT162b2 or mRNA-1273 primary vaccination series were also excluded. SARS-CoV-2 infection of the participants was determined by self-reporting a positive COVID-19 test (PCR or antigen) and/or anti-Nucleoprotein IgG antibody titers above 14.3 BAU/ml. Additionally, individuals with anti-Spike IgG antibody titers above 10.1 BAU/ml prior to primary vaccination were also defined as infected, as previously reported (20).

When studying effect of age for some analysis the study participants were divided in three different age groups (18-59, 60-69, and 70+ years), which were based on the age-related risk groups defined by the Dutch government that guided the COVID-19 booster vaccination strategy in the Netherlands. 

### Sample collection

Heparinized blood samples were collected from participants prior to vaccination (P0), 28 days post completion of the primary series (P28), prior to booster vaccination (B0) and 28 days post booster vaccination (B28) (**Figure 1A**). From all blood samples, peripheral blood mononuclear cells (PBMC) were isolated using Lymphoprep (Progen) and cryopreserved at −135°C.

### Synthetic peptides

Synthetic overlapping peptide pools covering the complete Spike protein from the different SARS-CoV-2 variants were all purchased from JPT; Ancestral (PM-WCPV-S-1), B.1.1.7 (Alpha; PM-SARS2-SMUT01-1), B.1.351 (Beta; PM-SARS2-SMUT02-1), P.1 (Gamma; PM-SARS2-SMUT03-1), B.1.617 (Delta; PM-SARS2-SMUT06-1), B.1.1.529 BA.1 (Omicron BA.1; PM-SARS2-SMUT08-1). All peptide pools contained 315 15-mers with 11 amino acid overlap. Peptide pools were reconstituted in 50µl DMSO and stored at -20°C until use according to the manufacturers’ instructions.

### IFNγ T cell ELISpot

IFNγ T cell ELISpot was performed as described before (21). PBMCs were thawed and plated in AIM-V medium (Gibco BRL, 12055-083) with 2% heat-inactivated human serum (Sigma, H6914) into each well, at 2x10^5^ cells/well (or 5x10^4^ cells/well for the positive control), of 96-well plates pre-coated with anti-human IFNγ antibodies (5 µg/ml, clone 1-D1K, Mabtech, 3420-3-1000). Subsequently, cells were stimulated in triplicate with DMSO (negative control), PHA (positive control; 1 µg/ml), or Spike peptide pools (0.65 µg/ml per peptide) for 22-24 hours (37°C, 5% CO_2_). Next, plates were washed with PBS+0.05% Tween 20 and incubated with anti-human IFNγ biotinylated antibody (1 μg/ml, 7-B6-1, MabTech, 3420-6-1000) in PBS+0.5% FBS for 1 hour. After washing with PBS+0.05% Tween20, the plates were incubated with Extravidin Alkaline Phosphatase (1 μg/ml; Sigma, E2636) in PBS+0.5% FBS for 1 hour. Subsequently, after washing with PBS+0.05% Tween20, the plates were washed with PBS and developed with TMB or 5-bromo-4-chloro-3-indolyl phosphate (BCIP)/nitro blue tetrazolium (NBT) (SigmaFast; Sigma, B5655). The reaction was stopped after approximately 7 minutes using H_2_0 and the plates were dried for 15 minutes. Spots were analyzed with CTL reader and software, and the number of spots from DMSO controls was subtracted from total spot numbers induced by antigen-specific stimulation. Samples that showed a low PHA response (<33 Spot Forming Units (SFU)/5x10^4^ PBMC) compared to other samples from the same donor were excluded from the analysis due to technical issues. Samples showing no response were calculated as 0.15 SFU/2x10^5^ PBMCs (representing half of the minimum of 1 spot per triplicate). Finally, results are reported as IFNγ SFU per 1x10^6^ PBMCs.

### Cytomegalovirus seropositivity

An in-house developed multiplex immunoassay was used to quantify anti-CMV IgG antibodies (22). Thresholds to determine CMV-seropositivity were adapted from a previous study (23). Individuals were categorized as seronegative if IgG concentration was <4 relative units (RU)/ml; as borderline if ≥4 RU/ml and < 7.5 RU/ml; and as seropositive if ≥7.5 RU/ml. Donors classified as borderline were excluded from analysis when CMV status was taken into consideration (see **Table 1**).

**Table 1:** Donor characteristics.

Characteristics	Primary vaccine				Total
	BNT162b2	mRNA-1273	ChAdOx1 nCoV-19	Ad26.COV2.S	
Donors (n)					
	125	71	3	9	208
Gender (n, %)					
Male	56 (44.8%)	35 (49.3%)	–	6 (66.7%)	97
Female	69 (55.2%)	36 (50.7%)	3 (100%)	3 (33.3%)	111
CMV status (n, %)					
Negative	53 (42.4%)	31 (43.6%)	1 (33.3%)	6	91
Positive	68 (54.4%)	39 (55%)	2 (66,7%)	3	112
Borderline	4 (3.2%)	1 (1.4%)	–	–	5
Age range (years)					
	19-99	22-79	64-64	18-49	
Age (years, mean ± SD)					
	55.5 ± 22.7	57 ± 15.7	64 ± 0	27.9 ± 10.3	
Age cohort (n, %)					
18-59	61 (48.8%)	38 (53.2%)	–	9 (100%)	108
60-69	19 (15.2%)	13 (18.3%)	3 (100%)	–	35
70+	45 (36%)	20 (28.2%)	–	–	65
Booster vaccine (n, %)					
BNT162b2	38 (48.1%)	34 (87.1%)	–	–	76
mRNA-1273	41 (51.9%)	5 (6.3%)	–	–	48

### Statistical analysis

Values below the detection limit of the ELISpot assay (0.75 SFU/10^6^ PBMCs) were recorded at 0.75 and missing data were excluded list-wise. Log_2_ of fold change (log_2_FC) was used to describe the changes in Spike-specific T cell ELISpot values between different timepoints. Data were visualized by means of boxplots, violin plots and linear trends with dots representing individual datapoints. In boxplots, median and interquartile range are provided.

For univariate hypothesis testing, figure legends specify the utilized statistical test. On the other hand, we employed linear models to establish multivariate associations between the longitudinal vaccine response, baseline characteristics (age, sex, CMV status), and the different vaccines received. Owing to the different vaccination regimes received at different timepoints, BNT162b2 or mRNA-1273 at P28 and B0; and BNT162b2/BNT162b2, BNT162b2/mRNA-1273, and mRNA-1273/BNT162b2 at B28, two separate models were required. To estimate the IFNγ T cell response to primary vaccination at timepoints P28 and B0, we established a mixed-effects regression model. As fixed effects, sex, an interaction term between age and CMV status, and an interaction term between timepoint and the type of primary vaccine were considered. As random effects, a random intercept per donor and a random slope per timepoint were defined (**Table 2**). On the other hand, we established a fixed-effects model to estimate the IFNγ T cell response to the different primary-booster vaccination regimes at B28, replacing the primary vaccine factor by the primary-booster vaccination regime (**Table 3**). Lastly, to evaluate the induction of the T cell response by the different primary-booster vaccine combinations and the effect of time in between the two vaccines, we established a fixed-effects model to estimate the log_2_FC between the IFNγ T cell response at B28 and at P28. To tackle the multi-collinearity issue between age and time to booster vaccination, these two variables were centralized around their mean. The interaction in between the resulting variables, sex, and the log_2_FC between the response at B0 and at P28 were defined as main effects (**Table 4**).

**Table 2:** Linear regression mixed model results for Spike-specific T cell response at timepoints P28 and B0.

	Estimate	95%CI	p-value
Intercept			
	4,459	3,559; 5,359	0,000
Age in years			
	-0,012	-0,027; 0,003	0,118
Sex			
Male	Ref.		
Female	0,164	-0,218; 0,545	0,398
CMV status			
Negative	Ref.		
Positive	1,298	0,113; 2,483	0,032
Timepoint			
P28	Ref.		
B0	-0,704	-1,075; -0,332	0,000
Primary vaccine			
BNT162b2	Ref.		
mRNA-1273	0,629	0,195; 1,063	0,005
Timepoint*Vaccine combination			
B0 * mRNA-1273	-0,889	-1,462; -0,316	0,003
Age in years*CMV status			
	-0,029	-0,048; -0,009	0,004

Conditional R^2^ = 0.312; marginal R^2^ = 0.926.

*indicates an interaction term; Ref. is reference; CI, confidence interval.

**Table 3:** Linear regression model results for Spike-specific T cell response at timepoint B28.

	Estimate	Standard Error	p-value
Intercept			
	4,793	0,555	0,000
Age (centralized)			
	-0,023	0,009	0,013
Sex			
Male	Ref.		
Female	0,610	0,214	0,005
CMV status			
Negative	Ref.		
Positive	1,794	0,728	0,015
Vaccine combination			
BNT162b2/BNT162b2	Ref.		
BNT162b2/mRNA-1273	1,134	0,272	0,000
mRNA-1273/BNT162b2	-0,113	0,269	0,676
Age (centralized)*CMV status			
	-0,035	0,012	0,004

R^2^ = 0.412.

*indicates an interaction term; Ref. is reference.

**Table 4:** Linear regression model results for the log2 fold change of B28/P28 Spike-specific T cell response.

	Estimate	Standard Error	p-value
Intercept			
	-0,155	0,359	0,667
Age (centralized)			
	0,000	0,018	0,996
Sex			
Male	Ref.		
Female	0,546	0,190	0,004
Months between primary-booster (centralized)			
	-0,255	0,299	0,394
Vaccine combination			
BNT162b2/BNT162b2	Ref.		
BNT162b2/mRNA-1273	0,711	0,279	0,011
mRNA-1273/BNT162b2	-0,726	0,367	0,049
CMV status			
Negative	Ref.		
Positive	0,910	0,252	0,000
Log fold change (B0/P28)			
	0,552	0,051	0,000
Months between primary-booster (centralized) * Age (centralized)			
	0,018	0,008	0,027
Months between primary-booster (centralized) * CMV status			
	0,296	0,299	0,324
Age (centralized) * CMV status			
	-0,020	0,018	0,258
Months between primary-booster (centralized) * Age (centralized) * CMV status			
	-0,038	0,010	0,000

R^2^ = 0.517.

*indicates an interaction term; Ref. is reference.

A p-value of <0.05 was regarded as statistically significant and data analysis and visualization were performed in RStudio software (R version 4.3.0, R Core Team).

## Results

### Participants and characteristics

We investigated COVID-19 vaccine-induced T cell responses in different age groups (range 18-99 years) of generally healthy adults in the Dutch population without history of SARS-CoV-2 infection(s) after their primary vaccination series and the first booster vaccination. The vaccination and sampling schemes are shown in **Figure 1A** and the characteristics of the different vaccine groups are given in **Table 1**. In total 125 participants received the BNT162b2 (Pfizer/BioNTech; two doses) vaccine for their primary series, 71 the mRNA-1273 (Moderna; two doses), 3 the ChAdOx1 nCoV-19 (AstraZeneca; two doses), and 9 the Ad26.COV2.S (Janssen; 1 dose) vaccine. Booster vaccination was followed up in a total of 81 BNT162b2- and 40 mRNA-1273-primed participants.

Age and sex composition was slightly different between the BNT162b2 and mRNA-1273 primary vaccinated groups, with the first composed of more younger female adults than the latter, yet not significantly (**Table 1**). Regarding CMV status, CMV seropositivity was significantly different across age groups in this dataset (p-value 0.0076; Kruskal-Wallis), but randomly occurring across sex and primary vaccination groups. The distribution of CMV-seropositive donors across age groups in this cohort was approximately 45% up to 69 years old and 70% among ≥70 years old donors, which reproduces the previously described CMV incidence in the general Dutch population (18, 19)).

### COVID-19 vaccines induce robust Spike-specific T cell responses upon primary vaccination, albeit lower responses in older adults

First, we evaluated the T cell responses induced after primary series with different COVID-19 vaccines (pre (P0) versus post (P28) vaccination). For this, the number of IFNγ-producing T cells was assessed by stimulating PBMCs with an overlapping peptide pool spanning the entire sequence of the Spike protein from the ancestral strain (**Figure 1B**). In the vast majority of the participants in all vaccine groups, a robust Spike-specific T cell response was induced (**Figure 1C**). Notably, higher T cell responses were induced by mRNA-1273 compared to BNT162b2 vaccination (**Figures 1C, D**).

When interrogating the effect of age in the vaccine-induced Spike-specific T cell response, we observed an inverse relation between the age of the vaccinees and the Spike-specific T cell frequency induced by primary vaccination in both BNT162b2 and mRNA1273 vaccine groups (**Figure 1D**). Stratifying participants according to CMV seropositivity and age groups (18-59, 60-69, and 70+ years), revealed that only in seropositive individuals ≥70 years old, this inverse relation was significant (**Figure 1E**). This is in line with the significant lower T cell frequencies in the 70+ versus the 18-59 age group for both BNT162b2- or mRNA1273-vaccinated CMV-seropositive individuals, although a trend in reduced levels in the 70+ age group is also found for CMV-seronegative participants in the mRNA1273-vaccinated group (**Figure 1F**). Of note, the higher responses induced by primary mRNA-1273 versus BNT162b2 vaccination was only observed in the CMV-seropositive 18-59 age group (**Figure 1E**). No significant effect of the sex of the vaccinees on the T cell response was found, even across age groups (**Supplementary Figures 1A, B**). Likewise, the inverse relation of Spike-specific T cell responses and age for CMV-seropositive donors was significant for both sexes, yet with a similar trend for CMV-seronegative males (**Supplementary Figure 1C**).

Together, these results indicate that all primary COVID-19 vaccine regimens induced a significant Spike-specific T cell response, with the mRNA-1273 vaccine inducing higher responses than the BNT162b2 vaccine mainly in the youngest adult age group (18-59). Age seems to negatively impact the height of the induced T cell response upon primary COVID-19 vaccination, which is most pronounced among CMV-seropositive older adults (70+).

### Waning of the Spike-specific T cell responses

**Figure 2:**
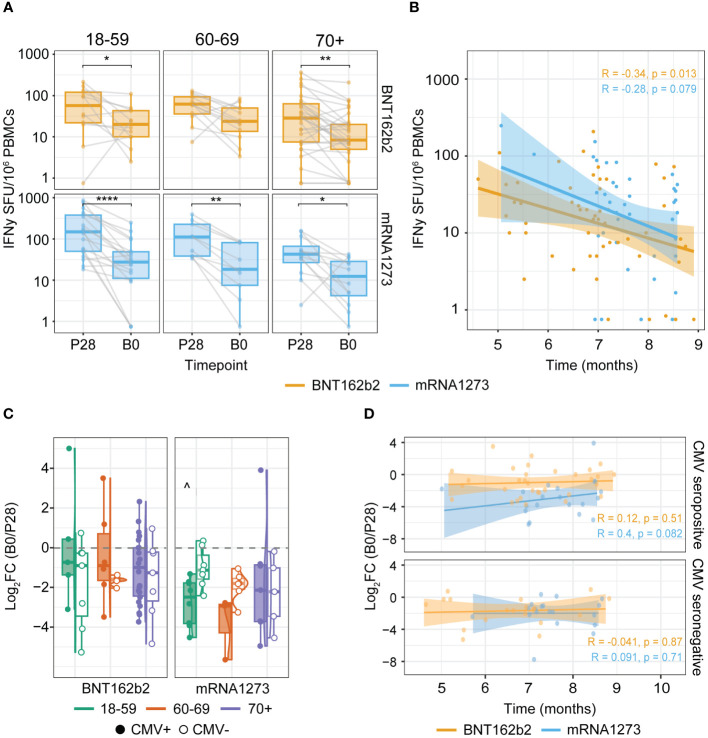
Spike-specific IFNγ T cell responses wane after primary vaccination but remain detectable up to at least nine months. **(A, B)** IFNγ T cell ELISpot response at timepoints P28 and B0 (1 and 4-9 months after completing the primary COVID-19 vaccination series respectively). **(C, D)** Change in the Spike-specific IFNγ T cell response between P28 and B0 defined by the log_2_ fold change (log_2_FC) of B0/P28. **(A)** Waning of Spike-specific T cell responses upon primary BNT162b2 or mRNA-1273 vaccination per age group. *p<0.05; **p<0.01; ****p<0.0001 paired Wilcoxon test. **(B)** Spearman correlation of the Spike-specific T cell response at B0 and the time in months between primary (second dose) and booster vaccination. **(C)** Log_2_FC of B0/P28 Spike-specific T cell response. ^p<0.05 BNT162b2 versus mRNA1273 among 18-59 cytomegalovirus (CMV) seropositive participants. Kruskal-Wallis test and Dunn’s test with Benjamin-Hochberg adjustment for multiple comparisons. **(D)** Spearman correlation of the log_2_FC of B0/P28 Spike-specific T cell response and time in months between the two vaccination dates stratified by CMV serostatus. Correlation coefficients (R) and p-values (p) are given according to the type of mRNA COVID-19 vaccine received (orange: BNT162b2; blue: mRNA-1273). SFU: Spot Forming Units.

Next, we investigated possible differences in the decline of the T cell response after primary vaccination across age groups and CMV status. For this purpose, we compared T cell responses at 28 days post primary series (P28) to pre-booster vaccination (B0). As expected, T cell frequencies were lower at the B0 compared to the P28 timepoint (**Figure 2A**), yet remained significantly higher than pre-vaccination (P0) levels in all age groups (data not shown). A similar tendency was seen when further stratifying the data by CMV status and primary-booster vaccination regimes (**Supplementary Figure 2A**). The interval between completion of the primary series and booster vaccination ranged between 4-9 months, which could affect the detected Spike-specific T cell frequencies at B0. We observed a weak inverse relation between the B0 T cell levels and the length of the interval between primary and booster vaccination for both BNT162b2 and mRNA-1273 vaccinees, albeit not significant for the latter (**Figure 2B**). Additionally, waning of the T cell response between BNT162b2- versus mRNA-1273-vaccinated participants and across the different age groups was similar, as the log_2_ of fold change (Log_2_FC) of the B0/P28 response was comparable. However, taking CMV status into account, the 18-59 age group showed more waning (i.e. lower Log_2_FC) in the mRNA-1273 versus BNT162b2 vaccine group only among CMV-seropositive participants (**Figure 2C**). Moreover, no correlation was apparent between the interval between primary and booster vaccination and the decline in the Spike-specific T cell response in both CMV-seropositive and -seronegative vaccinees (**Figure 2D**).

Together, this indicates that in all age groups the induced Spike-specific T cell response waned between 4 and 9 months after the primary series, but T cells were still detectable in the peripheral blood. This suggests maintenance of a Spike-specific memory T cell population that could be recalled when encountering the Spike antigen.

### Spike-specific T cell responses after COVID-19 booster vaccination

**Figure 3:**
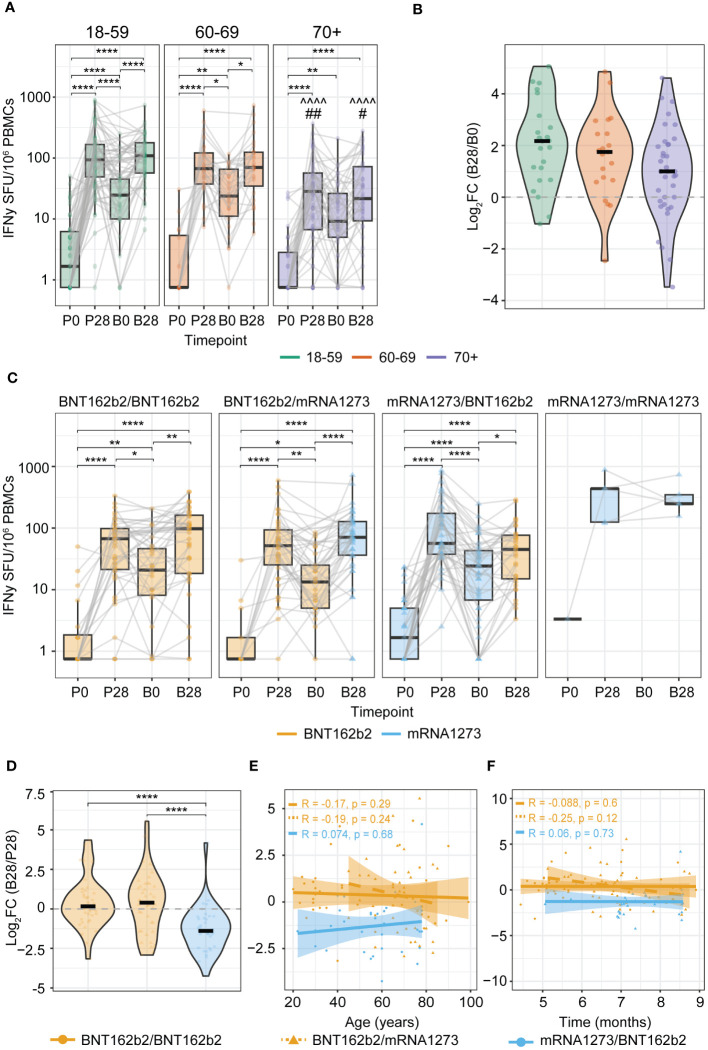
Increased levels of anti-Spike IFNγ T cell responses induced by booster vaccination. **(A, C)** IFNγ T cell ELISpot response before and after primary and booster vaccination stratified by: **(A)** Age group (^^^^^^p<0.0001 of 70+ versus same timepoint of 18-59 age group; #p<0.05, ## p<0.01 of 70+ versus same timepoint of 60-69 age group); **(C)** Primary-booster vaccine combination. *p<0.05,**p<0.01,****p<0.0001; Kruskal-Wallis test and Dunn’s test with Benjamini-Hochberg adjustment for multiple comparisons. Data points are colored according to the type of mRNA COVID-19 vaccine received at each timepoint (orange: BNT162b2; blue: mRNA-1273). P0: pre-vaccination; P28: 28 days post-primary vaccination series; B0: pre-booster vaccination; B28: 28 days post-booster vaccination. **(B)** Change in the Spike-specific IFNγ T cell response between B0 and B28 defined by the log2 fold change (log2FC) of B28/B0 stratified by age group. **(D)** Change in the Spike-specific IFNγ T cell response between P28 and B28 defined by the log_2_FC of B28/P28 stratified by primary-booster vaccine combination. **(E, F)** Spearman correlation of the log_2_FC of B28/P28 Spike-specific T cell response and: **(E)** Age; or **(F)** Time in months between primary (second dose) and booster vaccination. Correlation coefficients (R) and p-values (p) are given according to the primary-booster vaccine combination received (orange filled line: BNT162b2/BNT162b2; orange dashed line: BNT162b2/mRNA-1273, blue: mRNA-1273/mRNA-1273). SFU: Spot Forming Units.

Subsequently, we investigated to what extent a booster vaccine dose, given 4-9 months after completion of the primary series, increased the Spike-specific T cell frequencies. This revealed that for the 18-59 and 60-69 age groups, the Spike-specific T cell response was significantly elevated at 28 days upon booster vaccination (B28 versus B0), with a similar trend for the 70+ age group (**Figures 3A, B**). Additionally, the ultimate number of boosted IFNγ-producing T cells of the 70+ age group were significantly lower than those in the younger adult age groups (**Figure 3A**).

Differences were observed when interrogating the different primary-booster vaccination regimens. Both groups with BNT162b2 as a primary vaccine (i.e. BNT162b2/BNT162b2 and BNT162b2/mRNA-1273) showed a significant increase in the Spike-specific T cell response upon booster vaccination (B28 versus B0) (**Figure 3C** first and second panels), reaching similar levels as shown after the primary series at P28 (**Figure 3D**). In contrast, the mRNA-1273/BNT162b2 primary-booster combination resulted in a limited boosting effect as reflected by a negative log_2_FC, and strikingly, T cell frequencies even tended to be lower after booster vaccination (B28) than after the primary vaccination series (P28) (P28 versus B28, p-value 0.051; **Figures 3C, D**). This effect was not directly correlated with age (**Figure 3E**) nor with the time interval between primary and booster vaccination (**Figure 3F**). Notably, the ultimate boosted T cell frequencies at B28 were comparable between all primary-booster vaccine combination groups (**Figure 3C**). Moreover, CMV status did not affect these observations across any of the age groups (**Supplementary Figures 2A-E**).

Together, we gathered evidence that recovering the post-primary Spike-specific T cell response upon booster vaccination is affected by the given primary-booster vaccination regimen, with the mRNA-1273/BNT162b2 mounting limiting boosting effects.

### Multivariate analysis demonstrates additional effects of CMV seropositivity on Spike-specific T cell responses upon COVID-19 vaccination

We additionally applied a multivariate analysis to investigate the effect of age, sex and CMV status or their interactions on the vaccination induced Spike-specific T cell response. Importantly, these models confirmed the significant decrease of the Spike-specific T cell response with aging among CMV-seropositive donors (**Supplementary Figure 2F**, filled lines; **Tables 2**, **3**); the higher primary T cell response induced by mRNA-1273 compared to BNT162b2 (**Supplementary Figure 2F**, P28; **Table 2**); and the limited boosting capacity of the heterologous mRNA-1273/BNT162b2 vaccine combination independently of the time interval between vaccines (**Supplementary Figure 2G**, **Table 4**).

Conversely, these models also challenged previous observations made from non-parametric univariate analysis. For example, upon adjusting for age, sex, CMV status and the type of primary vaccine received, the estimated T cell response does not support the significant decrease with age independently of CMV status (**Table 2**). In addition, while no significant differences were seen when comparing the IFNγ T cell response between sexes, the results of the multivariate model depicted in **Table 3** estimated higher vaccine-induced T cell responses for females compared to males at B28. Likewise, despite no differences were found when comparing the T cell response at B28 in the two different BNT162b2-primed vaccine groups, the model estimates a higher response for donors receiving the BNT162b2/mRNA-1273 versus the BNT162b2/BNT162b2 primary-booster vaccination regimen. Owing to the poor fitness of both models and potential confounding, evidenced by the low R^2^ values depicted in **Tables 2**–**4**, it should be noted that there might be other more informative variables not considered in the present study that could improve the longitudinal estimation of the T cell response to COVID-19 vaccination.

### T cell cross-reactivity to other SARS-CoV-2 variants

**Figure 4:**
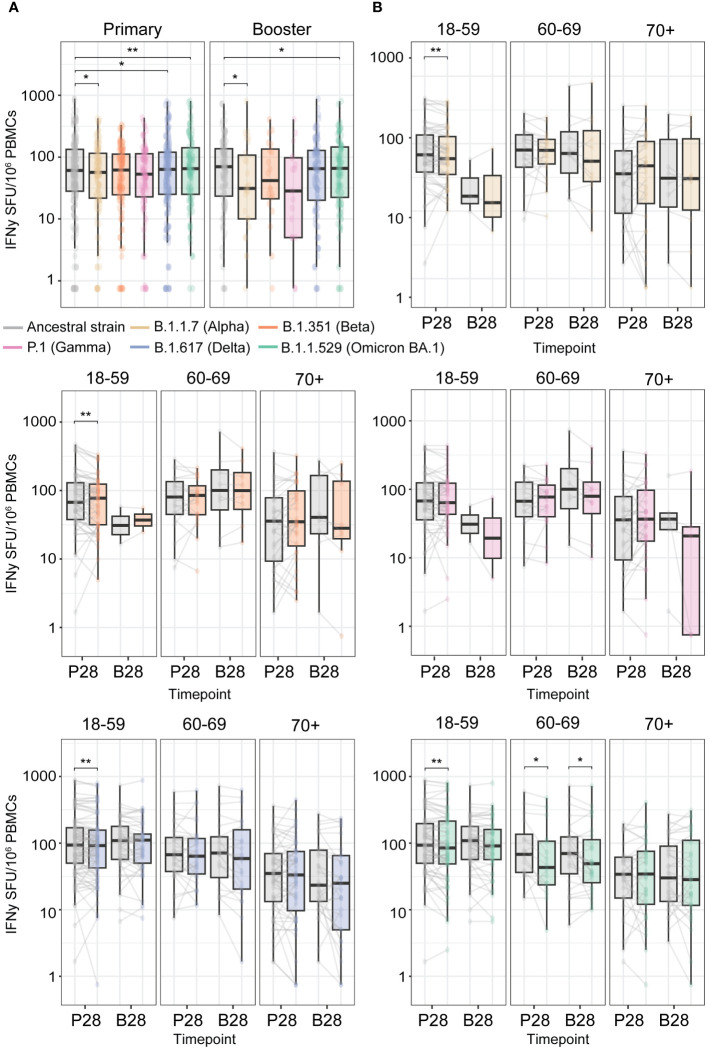
High cross-reactivity of primary and booster vaccination-induced anti-Spike IFNγ T cell responses with the Spike protein of Variants of Concern (VOCs). T cell responses were measured at P28 and B28 using an IFNγ T cell ELISpot assay upon stimulation with an overlapping peptide pool of the Spike protein from the ancestral SARS-CoV2 vaccine strain and from the different VOCs. **(A)** Comparison of the Spike-specific T cell response from the ancestral strain to different VOCs. **(B)** Per VOC comparison to the ancestral strain Spike-specific T cell response stratified per age group. *p<0.05; **p<0.01 paired Wilcoxon test.

During the COVID-19 pandemic, multiple VOCs emerged. Several studies indicate that vaccination-induced T cell responses are highly cross-reactive to the Spike proteins of SARS-CoV-2 variants (3, 5–8). To assess cross-reactivity, we performed a paired analysis just for those participants who showed an IFNγ T cell response against the Spike protein from the ancestral strain above the detection limit of the assay (>0.75 SFU/10^6^ PBMCs). Across the whole cohort, we found a slight reduction in cross-reactivity to the Spike protein of Alpha (B.1.1.7), Delta (B.1.617), and Omicron BA.1 (B.1.1.529 BA.1) versus the ancestral variant after primary vaccination. This effect was similar upon booster vaccination, except for the Delta variant (**Figure 4A**). Taking age into account, the reduction in reactivity to Alpha, Delta, as well as Beta was seen in the 18-59 age group after primary vaccination (P28), while for Omicron BA.1, it was observed in both the 18-59 and 60-69 age groups (**Figure 4B**). After booster vaccination (B28), the reduction in the T cell response was only observed for Omicron BA.1 (B.1.1.529 BA.1) in the 60-69 age group. No significant reduction to all tested VOCs was found for the 70+ age group (**Figure 4B**), which might be a consequence of the higher variation in the T cell response observed in this group. Additionally, the observed reduction in cross-reactivity was diminished upon booster vaccination, indicating the capacity of a third vaccination to boost vaccine-induced cross-reactive T cells against VOCs. CMV seropositivity of the vaccinees did not affect cross-reactivity of vaccine-induced T cells within any of the age groups (**Supplementary Figure 3**), and similar patterns were observed across different primary-booster vaccine combinations (data not shown).

Overall, this indicates that there is only a small reduction in the cross-reactivity of ancestral vaccine-induced Spike-specific T cells against Alpha, Beta, Delta, and Omicron BA.1 variants. Booster vaccination seems to be required to generate a similar Spike-specific T cell response towards the vaccine ancestral strain and the emerging VOCs.

## Discussion

Our study showed that across all adult ages ranging from 18 to 99 years, Spike-specific T cells, regarded as important players in limiting COVID-19 disease severity (9–11) and hospitalization (6, 11, 12), were detectable after the primary COVID-19 vaccination series. Vaccination with mRNA-1273 induced the highest T cell responses in the population <60 years of age. However, we observed an inverse relationship with the age of the vaccinees as well as more variation in the T cell response of the older adult age group (70+ years). Strikingly, this observation could completely be attributed to a CMV-seropositive status of the vaccinees. Additionally, and independently of CMV seropositivity, IFNγ-producing T cell frequencies in the 70+ age group persisted to be the lowest compared with the other age groups, although booster vaccination generally increased the T cell response to post-primary levels across all ages. The primary-booster vaccine type combination also affected the Spike-specific T cell response measured after the booster dose.

In the Netherlands, primary vaccination was performed using four different vaccines (i.e. BNT162b2, mRNA-1273, ChAdOx1 nCoV-19, and Ad26.COV2.S). Here we show that all four vaccine formulations induced robust Spike-specific T cell frequencies upon primary vaccination. When comparing both mRNA-based vaccines, the Spike-specific T cell response was higher after mRNA-1273 versus BNT162b2 vaccination in the younger population (<60 years), which is comparable to what has been previously described (24). This observed elevated T cell response might be attributed to a higher antigen dose present in the mRNA-1273 vaccine used for primary vaccination (100 µg versus 30 µg mRNA in BNT162b2). The influence of dose on the strength of the Spike-specific T cell response was already demonstrated by comparing the standard mRNA-1273 vaccine containing 100 µg to one with 25 µg of mRNA in a dose finding study, with the highest dose inducing approximately a 1.4 to 2-fold higher Spike-specific CD4^+^ T cell response (25).

Induction of SARS-CoV-2-specific T cells upon vaccination is specifically important in older adults, who represent a vulnerable group with regard to COVID-19 (13). However, we, and others (26, 27), found a lower SARS-CoV-2-specific T cell response induced by primary vaccination in participants ≥70 years of age compared to younger adults. This might reflect a reduced capacity of older individuals to mount *de novo* T cell responses, as was previously shown upon primary tick-borne encephalitis (28), yellow fever vaccination (29), and using multiple *in vitro* approaches in the context of melanoma (30) and SARS-CoV-2 (31). For the latter, others demonstrated an age-related decline in the number of specificities of *in vitro*-generated *de novo* CD8^+^ T cells to a range of typically immunodominant SARS-CoV-2 epitopes (31). It has been hypothesized that this, amongst others, could be attributed to a lower abundance of naïve T cells (28, 30), a possible limited TCR repertoire of the naïve T cell compartment (30, 32), and impaired TCR signaling (30, 33). Additionally, dendritic cell numbers and/or functionality have been described to be compromised with ageing (29, 34).

Persistent latent viral infections such as with CMV have been suggested to exacerbate age-related modifications of adaptive immunity. Previous reports claim that it can lower the expression of the co-stimulatory molecule CD28 on naïve T cells and steer the naïve T cell repertoire towards a more differentiated phenotype (e.g. CD28^-^ effector memory or effector-memory re-expressing CD45RA (EMRA) T cells) with increased expression of senescence markers CD57 and KLRG-1 (35). Hence, latent CMV infection could hamper the mounting of *de novo* T cell responses, as was found in the study of Nicoli et al., in which upon primary tick-borne encephalitis vaccination, CMV-seropositive individuals had lower antigen-specific T cell frequencies (36). Literature regarding the effect of CMV status on the cellular response to COVID-19 vaccination is conflicting. For the younger adult age group (<60 years), we did not observe a significant effect of CMV status, although there is a clear trend towards a higher Spike-specific T cell response in seropositive mRNA-1273-vaccinated individuals. This is in line with findings of the study of Sharpe and colleagues in a cohort of ChAdOx1 nCoV-19-vaccinated young adults (<55 years) (37). On the other hand, our study shows that for older adults (≥70 years), the Spike-specific T cell response diminishes with increasing age only in the CMV-seropositive vaccinees. Additionally, the observed lower responsiveness to the primary COVID-19 mRNA vaccination of older adults compared to younger adults was more pronounced in CMV-seropositive individuals, yet only in the BNT162b2 vaccinated group. However, rather than being driven by the vaccine type, it might be attributed to a higher number of older (>79 years) CMV-seropositive participants with diminished T cell responses in this vaccine cohort. Conversely, a study focused on older adults (>80 years) found that after one dose of the BNT162b2 vaccine, CMV-seropositive donors triggered higher Spike-specific IFNγ T cell responses compared to CMV-seronegative participants (27). Whereas, others have shown no effect of CMV seropositivity on the height of the SARS-CoV-2-specific T cell response in infection-naïve older adults (>80 years) upon two or three doses of the BNT162b2 or mRNA-1273 vaccines (38).

Although we observed no effect of CMV status on the recall response, the responses in the 70+ age group persisted to be the lowest after booster vaccination. Whether additional booster vaccinations would eventually increase Spike-specific T cell frequencies remains to be tested. However, in immunocompromised allogenic stem cell transplantation recipients, administration of a second booster was found to increase T cell responses to similar levels as in healthy controls (39). Yet, whether this holds true for the healthy older adult population remains to be determined.

When investigating the T cell response to the different primary-booster vaccination regimens in our cohort, we found a beneficial effect of a heterologous strategy in BNT162b2-primed individuals (BNT162b2/mRNA-1273), as the T cell response after the mRNA-1273 vaccination was elevated post-booster (B28) compared to the homologous regimen when corrected for age, sex and CMV status in the multivariate analysis. This was corroborated by a study showing that a mRNA-1273 booster dose (50 µg) after three BNT162b2 vaccine doses enhanced cellular responses to a greater extent than four doses of BNT162b2 (40). However, this beneficial effect of the heterologous mRNA-1273 second booster in BNT162b2-primed and boosted individuals was not maintained, as two and three months after the second booster, the T cell response dropped to similar levels as in the homologous BNT162b2-boosted individuals (40).

Strikingly, we observed that individuals primed with mRNA-1273 receiving a BNT162b2 booster failed to recall the post-primary T cell response and tended to have lower post-booster responses than individuals receiving a BNT162b2/BNT162b2 or a BNT162b2/mRNA-1273 regimen. Due to the low number of individuals with a homologous mRNA-1273 primary-booster strategy, we could not assess if the reduced booster effect was due to the heterologous mRNA-1273/BNT162b2 schedule or a distinct priming by mRNA-1273. Whether this limited boosting effect in the mRNA-1273/BNT162b2 primary-booster vaccine group is maintained upon additional exposures needs to be investigated. However, several studies have shown that, in contrast to antibody levels (20, 41, 42), the Spike-specific T cell response reached a plateau and levels did not increase upon further exposure through booster immunizations (41) or infections (42). Thus, the observed higher Spike-specific T cell frequencies induced by mRNA-1273 primary vaccination might already induce a response reaching this plateau and limiting subsequent booster effects.

Vaccine effectiveness is hampered by the emergence of VOCs with antibody-escape mutations, resulting in reduced cross-neutralization of antibodies. In contrast, a study interrogating responses in convalescent adults (<70 years) found an average of 84.5% of the CD4^+^ and 95.3% of the CD8^+^ T cell epitopes of the Spike protein that are conserved between the ancestral strain and VOCs (Alpha, Beta, Gamma, and Epsilon) (43). Yet other studies did show a more significant loss of functional T cell responses to specific epitopes of Omicron variants (44, 45). We, and others (3, 5–8), show only a slight reduction in cross-reactivity of vaccination-induced Spike-specific T cells with the Spike of other VOCs (i.e. Alpha, Delta, and Omicron BA.1). We found that this decrease was mostly diminished upon booster vaccination. Notably, we did not observe a significant reduction to all tested VOCs for the 70+ age group. However, this might be due to the higher variation and overall lower responses found in this age group, making it harder to detect a potential reduction in cross-reactivity. Alternatively, older adults have also been described to mount antigen-specific CD8^+^ T cells towards a lower number of epitopes compared to younger adults upon stimulation *in vitro* with a range of typically immunodominant SARS-CoV-2 epitopes (31). Moreover, the older adults show a different immunodominance for the recognized epitopes. This could suggest that the epitopes recognized by older adults might be less prone to escape mutations. Still, interrogation of cross-reactivity of vaccine-induced responses to other VOCs would require a larger cohort of older adults to pinpoint whether there is indeed no loss of recognition in this age group.

This study had some limitations. First, Spike-specific responses were interrogated using a IFNγ ELISpot upon stimulation of PBMCs, an assay in which it cannot be distinguished which cells produce the cytokine. However, the fact that other studies using a flow cytometry read-out are in accordance with our findings [e.g. lower responses in older adults (26, 27)] suggests that our results are reliable. Nevertheless, investigation of other functionalities, such as production of other cytotoxic cytokines, and additional phenotyping analysis would be informative. Second, owing to the limited number of participants vaccinated with the vector-based vaccines (ChAdOx1 nCoV-19, and Ad26.COV2.S), extensive comparisons with the mRNA vaccinees was not possible. Finally, with the aim to study the vaccination-induced SARS-CoV-2-specific T cell response, our study was only focused on infection-naïve individuals. Although at present, a substantial part of the general population has developed hybrid immunity due to infection before, during or after vaccination. Yet, only a minority of the older adults (≥70 years) in our study had been infected at the time of the booster vaccination.

To conclude, this study showed that robust T cell responses were induced in all age groups upon primary vaccination with the different COVID-19 vaccines, with higher levels triggered by the mRNA-1273 vaccine in the younger population (18-59 years). Booster vaccination reinvigorated the post-primary T cell response, although a heterologous vaccination regime comprising a primary mRNA-1273 inoculation followed by a BNT162b2 booster vaccination seemed suboptimal. In older adults (≥70 years), the induction of the T cell response upon both primary and booster vaccination was overall lower and with higher variability, although their cross-reactive capacity to VOCs was maintained. The presence of cross-reactive T cells against new VOCs in all age groups could indicate (partial) protection against severe disease caused by new emerging variants. Lastly, CMV seropositivity showed distinct effects in younger versus older adults, as it seemed to be associated with increased and decreased primary vaccination-induced T cell responses respectively.

## Data availability statement

The raw data supporting the conclusions of this article will be made available by the authors, without undue reservation.

## Ethics statement

The studies involving humans were approved by Medical Research Ethics Committee Utrecht. The studies were conducted in accordance with the local legislation and institutional requirements. The participants provided their written informed consent to participate in this study. Clinical trial identifiers: COVID-19 vaccination in 18 to 60 years of age (NL76440.041.21, EudraCT: 2021-001357-31); in the aging population over 60 years of age (NL74843.041.21, EudraCT: 2021-001976-40); Vital (NL69701.041.19, EudraCT: 2019-000836-24).

## Author contributions

JB: Conceptualization, Formal analysis, Methodology, Visualization, Writing – original draft, Writing – review & editing. SS: Formal analysis, Visualization, Writing – original draft, Writing – review & editing. LdR: Investigation, Methodology, Writing – review & editing. MBvM: Investigation, Methodology, Writing – review & editing. PM: Investigation, Writing – review & editing. EvW: Investigation, Writing – review & editing. DO: Data curation, Writing – review & editing. LB: Data curation, Supervision, Writing – review & editing. NR: Conceptualization, Writing – review & editing. JvB: Conceptualization, Data curation, Writing – review & editing. MN: Formal analysis, Visualization, Writing – review & editing. CvE: Conceptualization, Writing – review & editing. MB: Conceptualization, Writing – review & editing. PK: Writing – review & editing. AB: Conceptualization, Funding acquisition, Methodology, Writing – review & editing. JdW: Conceptualization, Funding acquisition, Methodology, Writing – review & editing.
