# Supplementary material for: Distinct T cell responsiveness to different COVID-19 vaccines and cross-reactivity to SARS-CoV-2 variants with age and CMV status
Source: Front Immunol. 2024 May 7;15:1392477. doi: 10.3389/fimmu.2024.1392477 (PMC11106399; doi:10.3389/fimmu.2024.1392477)
Supplement: Supplementary file 1 [file DataSheet_1.docx]

*Distinct T cell responsiveness to different COVID-19 vaccines and cross-reactivity to SARS-CoV-2 variants with age and CMV status*

**J. Brummelman^1^*, S. Suárez-Hernández^1^, L. de Rond^1^, M. Bogaard-van Maurik^1^, P. Molenaar^1^, E.E. van Wijlen^1^, D. Oomen1, L. Beckers^1^, N.Y. Rots^1^, J. van Beek^1^, M. A. Nicolaie^1^, C.A.C.M. van Els^1,2^, M.C. Boer^1^, P. Kaaijk^1^, A.M. Buisman^1^, J. de Wit^1^**

*^1^Center for Infectious Disease Control, Dutch National Institute for Public Health and the Environment (RIVM), Bilthoven, The Netherlands*

*^2^Infectious Diseases and Immunology, Department of Biomolecular Health Sciences, Faculty of Veterinary Medicine, Utrecht University, Utrecht, The Netherlands*

Correspondence: [jolanda.brummelman@rivm.nl](mailto:jolanda.brummelman@rivm.nl)

# Supplementary Figures and Tables

## Supplementary Figures


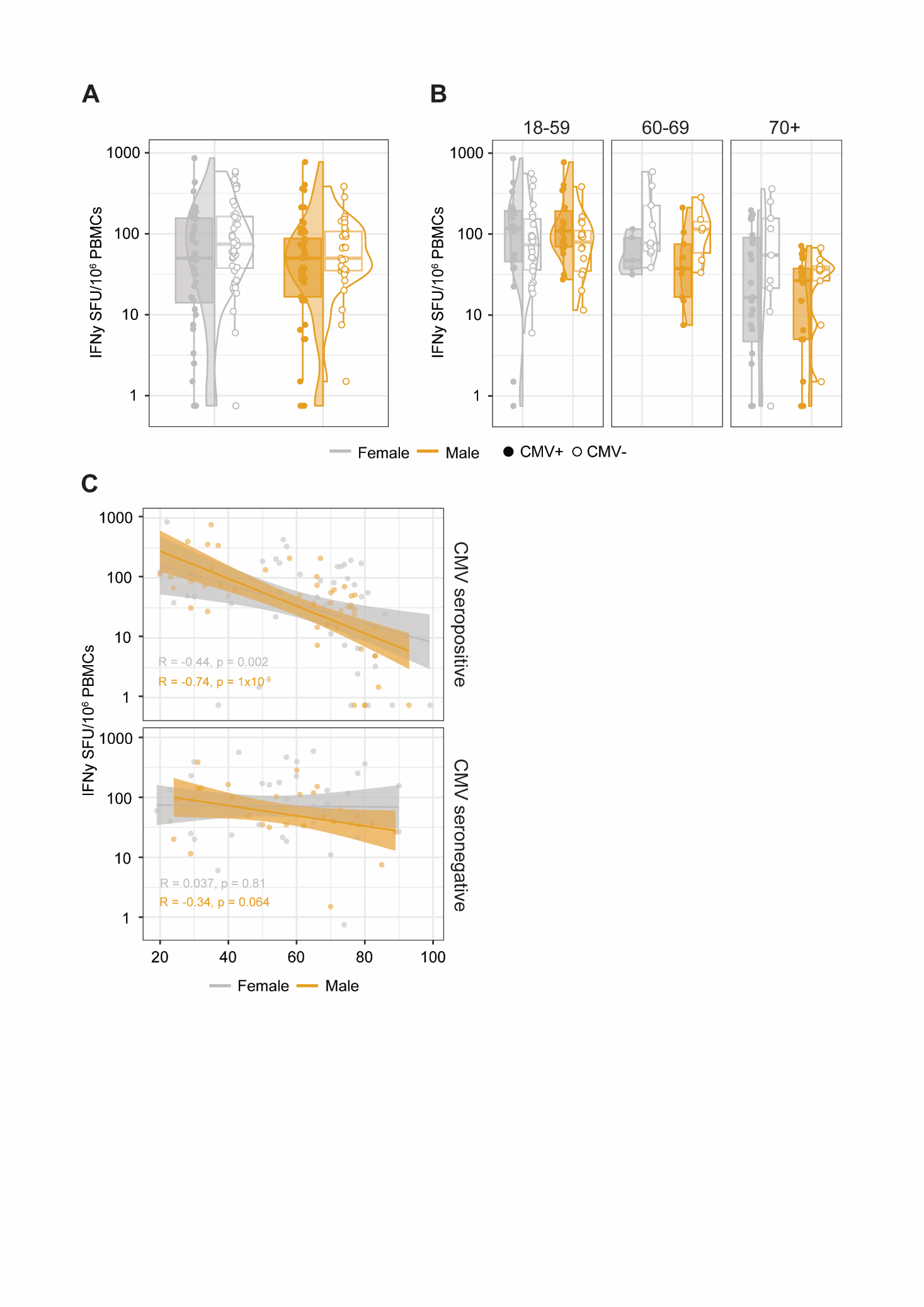
 **Supplementary Figure 1. Limited effect of sex on the anti-Spike T cell response induced by primary COVID-19 vaccination.** T cell responses were measured using an IFNγ T cell ELISpot upon stimulation with an overlapping peptide pool of the ancestral vaccine strain Spike protein. A-B) Spike-specific IFNγ T cell response at P28 stratified by: A) Cytomegalovirus (CMV) serostatus and sex; B) CMV status, sex and age group of the participants. C) Spearman correlation between age of the participants and the Spike-specific IFNγ T cell response at timepoint P28. Correlation coefficients (R) and p-values (p) are given per sex of the vaccinees (grey: female; orange: male). SFU: Spot Forming Units.


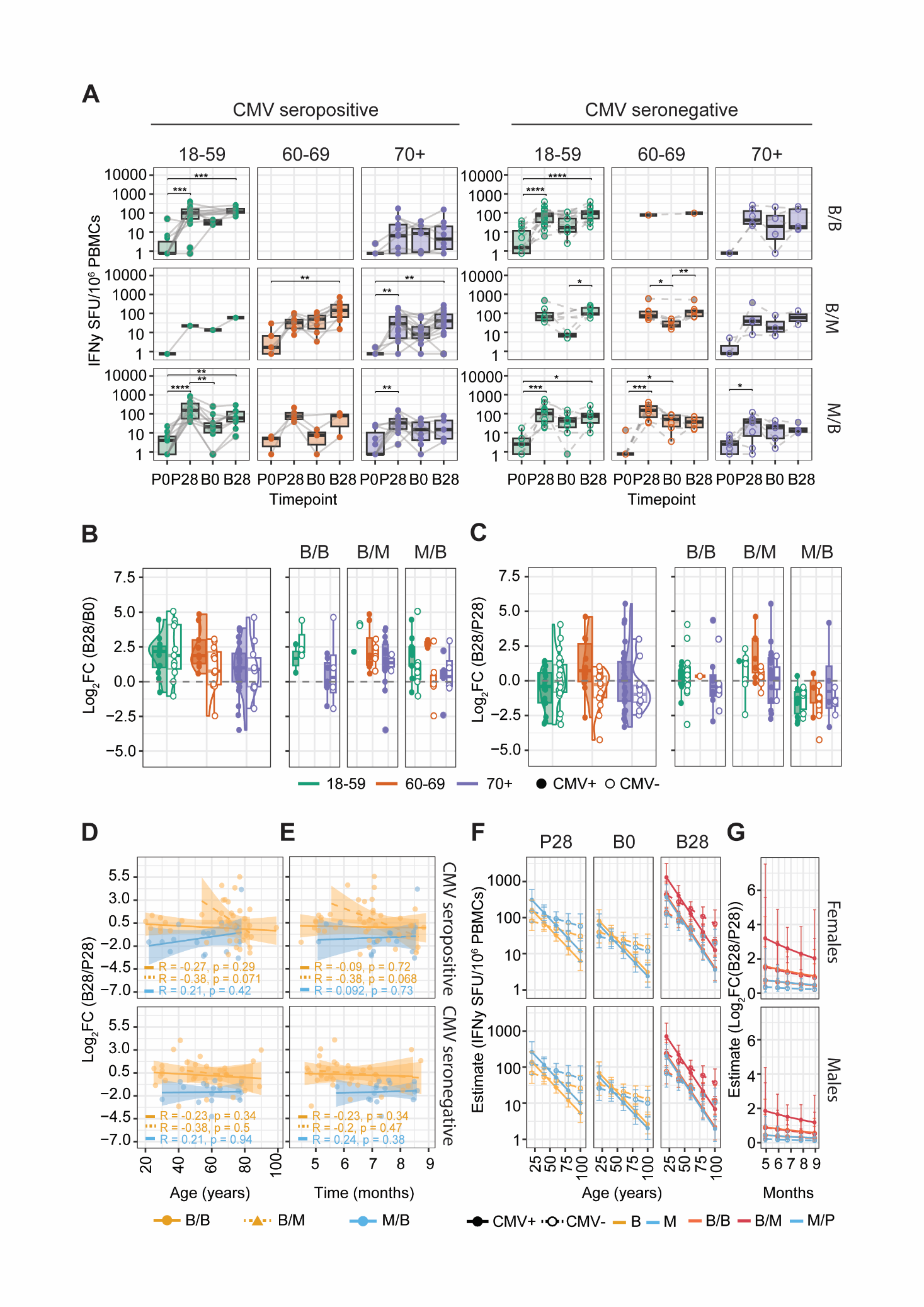


**Supplementary Figure 2. Limited booster effect observed in mRNA-1273/BNT162b2 vaccinated individuals.** A) Spike-specific T cell response before and after primary and booster vaccination stratified by cytomegalovirus (CMV) serostatus, age group and primary-booster vaccine combination. *p<0.05; **p<0.01, ***p<0.001,****p<0.0001 Kruskal-Wallis test and Dunn’s test with Benjamini-Hochberg adjustment for multiple comparisons. P0: pre-vaccination; P28: 28 days post-primary vaccination series; B0: pre-booster vaccination; B28: 28 days post-booster vaccination. B-C) Change in the Spike-specific IFNγ T cell response defined by the log_2_ fold change (log_2_FC) between: B) B0 and B28; or C) P28 and B28 stratified by CMV status, age group and primary-booster vaccine combination. D-E) Spearman correlation of the log_2_FC of B28/P28 Spike-specific T cell response and: D) Age; or E) Time in months between primary (second dose) and booster vaccination. Correlation coefficients (R) and p-values (p) are given according to the primary-booster vaccine combination received (orange filled line: BNT162b2/BNT162b2; orange dashed line: BNT162b2/mRNA-1273, blue: mRNA-1273/mRNA-1273). F) Estimates of the Spike-specific T cell response across post-vaccination timepoints stratified by sex, CMV status and primary vaccine (P28, B0) or primary-booster vaccine combination (B28). P28 and B0 estimates belong to the mixed-effects linear model defined in Table 2 while the estimates of B28 were defined by the fixed-effects linear model depicted in Table 3. G) Estimates of the booster-induced recovery of the primary vaccination T cell response expressed as the log_2_FC of B28/P28 and defined by the fixed-effects linear model depicted in Table 4. SFU: Spot Forming Units. B: BNT162b2. M: mRNA-1273.


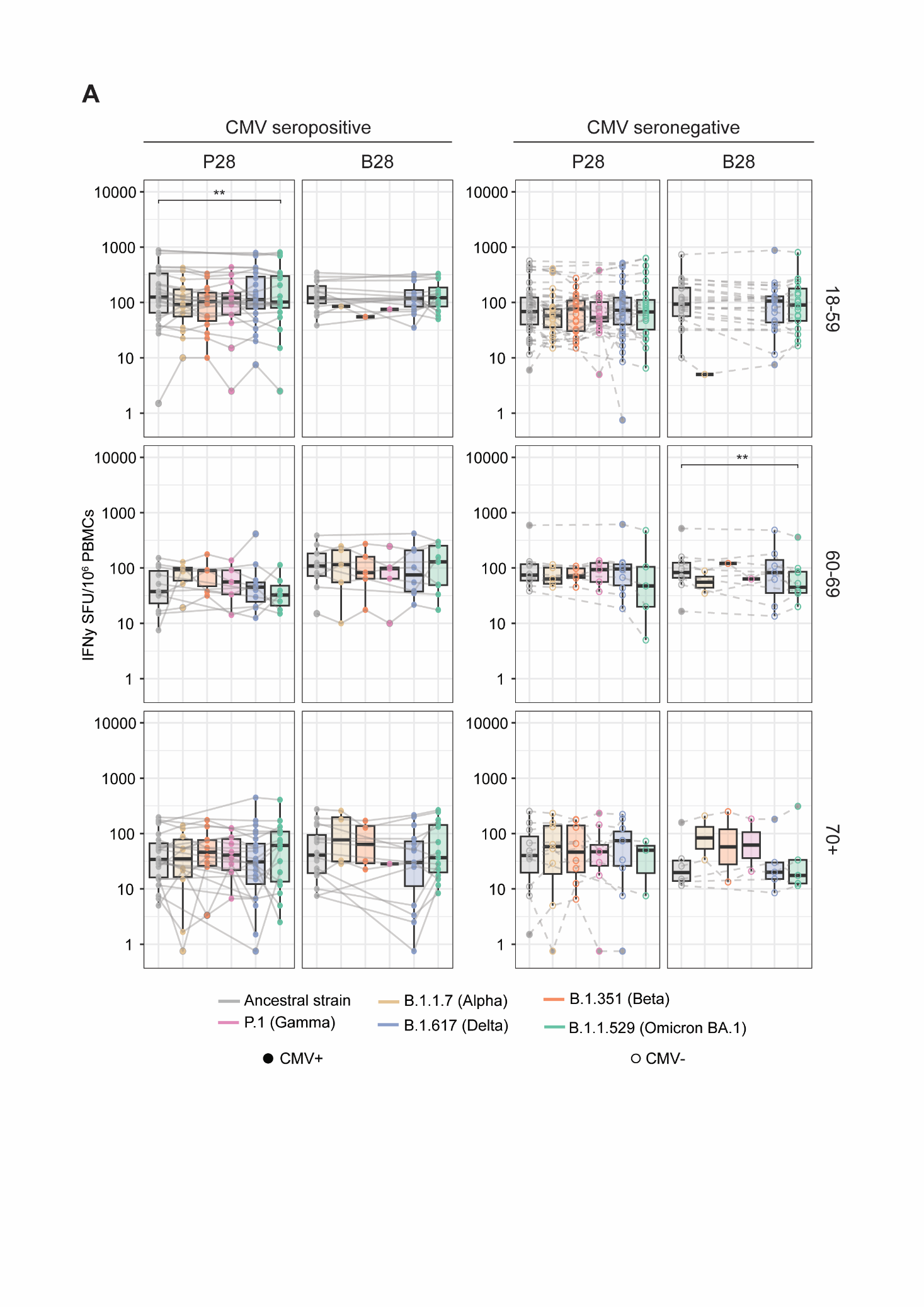


**
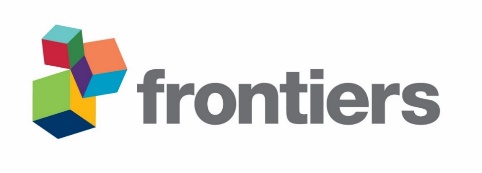
Supplementary Figure 3. Cross-reactivity of primary and booster vaccination-induced anti-Spike T cell responses with Spike protein of** **Variants of Concern (VOCs)**. The frequencies of T cells specific for the Spike protein of the different VOCs were measured at P28 and B28 using distinct VOC overlapping peptide pools as stimulation in an IFNγ T cell ELISpot. Shown are the comparison of the T cell frequencies in paired-samples of the ancestral vaccine variant versus the Alpha (B.1.1.7), Beta (B.1.351), Gamma (P.1), Delta (B.1.617) variants at the P28 and B28 stratified by cytomegalovirus (CMV) serostatus, timepoint and age cohort. *p<0.05; **p<0.01 paired Wilcoxon test.
